# Cigarette Smoke Affects Dendritic Cell Populations, Epithelial Barrier Function, and the Immune Response to Viral Infection With H1N1

**DOI:** 10.3389/fmed.2020.571003

**Published:** 2020-11-06

**Authors:** Olga Danov, Martin Wolff, Sabine Bartel, Sebastian Böhlen, Helena Obernolte, Sabine Wronski, Danny Jonigk, Barbara Hammer, Draginja Kovacevic, Sebastian Reuter, Susanne Krauss-Etschmann, Katherina Sewald

**Affiliations:** ^1^Fraunhofer Institute for Toxicology and Experimental Medicine ITEM, Biomedical Research in Endstage and Obstructive Lung Disease Hannover (BREATH), Member of the German Center for Lung Research (DZL), Member of Fraunhofer International Consortium for Anti-Infective Research (iCAIR), Member of Centre for Immune Mediated Diseases (CIMD), Hanover, Germany; ^2^Early Origins of Chronic Lung Diseases, Priority Area Asthma and Allergy, Research Center Borstel – Leibniz Lung Center, Airway Research Center North (ARCN), Member of the German Center for Lung Research (DZL), Borstel, Germany; ^3^Department of Pathology and Medical Biology, University Medical Center Groningen, GRIAC Research Institute, University of Groningen, Groningen, Netherlands; ^4^Department of Pathology, Hannover Medical School, Biomedical Research in Endstage and Obstructive Lung Disease Hannover (BREATH), Hanover, Germany; ^5^Department of Pulmonary Medicine, University Medical Center Essen – Ruhrlandklinik, Essen, Germany; ^6^Asthma Research, Institute of Experimental Medicine, Christian-Albrechts-Universität zu Kiel, Kiel, Germany

**Keywords:** cigarette smoke exposure, dendritic cells, epithelial barrier, influenza virus, mouse model, precision-cut lung slice

## Abstract

Smokers with apparently “healthy” lungs suffer from more severe and frequent viral respiratory infections, but the mechanisms underlying this observation are still unclear. Epithelial cells and dendritic cells (DC) form the first line of defense against inhaled noxes such as smoke or viruses. We therefore aimed to obtain insight into how cigarette smoke affects DCs and epithelial cells and how this influences the response to viral infection. Female C57BL/6J mice were exposed to cigarette smoke (CS) for 1 h daily for 24 days and then challenged i.n. with the viral mimic and Toll-like receptor 3 (TLR3) ligand poly (I:C) after the last exposure. DC subpopulations were analyzed 24 h later in whole lung homogenates by flow cytometry. Calu-3 cells or human precision-cut lung slices (PCLS) cultured at air-liquid interface were exposed to CS or air and subsequently inoculated with influenza H1N1. At 48 h post infection cytokines were analyzed by multiplex technology. Cytotoxic effects were measured by release of lactate dehydrogenase (LDH) and confocal imaging. In Calu-3 cells the trans-epithelial electrical resistance (TEER) was assessed. Smoke exposure of mice increased numbers of inflammatory and plasmacytoid DCs in lung tissue. Additional poly (I:C) challenge further increased the population of inflammatory DCs and conventional DCs, especially CD11b^+^ cDCs. Smoke exposure led to a loss of the barrier function in Calu-3 cells, which was further exaggerated by additional influenza H1N1 infection. Influenza H1N1-induced secretion of antiviral cytokines (IFN-α2a, IFN-λ, interferon-γ-induced protein 10 [IP-10]), pro-inflammatory cytokine IL-6, as well as T cell-associated cytokines (e.g., I-TAC) were completely suppressed in both Calu-3 cells and human PCLS after smoke exposure. In summary, cigarette smoke exposure increased the number of inflammatory DCs in the lung and disrupted epithelial barrier functions, both of which was further enhanced by viral stimulation. Additionally, the antiviral immune response to influenza H1N1 was strongly suppressed by smoke. These data suggest that smoke impairs protective innate mechanisms in the lung, which could be responsible for the increased susceptibility to viral infections in “healthy” smokers.

## Introduction

Cigarette smoking is a leading cause of morbidity and mortality accounting for around 18% of all deaths worldwide (1). It is the main risk factor for chronic obstructive pulmonary disease and lung cancer and furthermore leads to more frequent and severe bacterial and viral respiratory tract infections (2–5). Cigarette smoking impairs ciliary function, increases mucus secretion, and leads to an influx of inflammatory cells such as macrophages and neutrophils into the respiratory tract. These events are likely to alter the respiratory tract's ability to defend itself from infection (6, 7).

Influenza virus is a common human respiratory pathogen causing seasonal epidemics and pandemic outbreaks, such as the most recent one with influenza H1N1 in 2009. Epidemiological studies have shown that smokers are more susceptible to influenza virus infections than non-smokers and suffer from more severe symptoms resulting in higher influenza-related mortality (2, 6, 8–11). Mechanistically, the available evidence suggests that the effects of cigarette smoke (CS) on susceptibility to influenza infection include the host's early innate defense against viruses (12).

Upon viral infection, the first rapid response is provided by the respiratory innate immune system. This defense system is built by the epithelial layer and resident dendritic cells (DC) that are in close contact with each other. In respiratory viral infection, lung DCs migrate to mediastinal lymph nodes to generate adapted cytotoxic T cell responses (7, 13, 14).

The primary target of viral replication is the pulmonary epithelium as influenza virus mainly infects airway and alveolar epithelial cells. Upon infection, they secrete IFNs, thereby recruiting so-called inflammatory DC (iDC) to the lung. iDCs either immigrate from the bone marrow or arise from transformed monocytes (15, 16). Vice versa, pDCs secrete type I and III interferons, which upregulate the expression of class I major histocompatibility complex (MHC) on epithelial cells, thereby promoting an antiviral response (17, 18).

Based on surface markers, resident DCs are classified into conventional DC (cDC) and plasmacytoid DC (pDC), both of which play a key role in immune surveillance in the lung (7, 19). cDC are further subdivided into crosspresenting CD103^+^ cDC and CD11b^+^ cDC (7, 20), which trigger adaptive CD8 and CD4 T cell and B cell responses. CD103^+^ and CD11b^+^ migratory cDCs were shown to mediate cytotoxic T cell (CTL) response against influenza A virus infection, while the absence of CD103^+^ migratory cDCs has led to significantly increased mortality with lack of viral clearance in mice (21, 22). CD11b expressing cDCs are associated with inflammatory stimuli and are present in the respiratory tract during activation of the immune response (23–25). CD103 positive cDCs, on the other hand, are resident and occur in the vicinity of mucous membranes and respiratory epithelium (26).

At the molecular level, viral pathogen-associated molecular patterns are recognized by pathogen recognition receptors (PRRs) such as the Toll-like receptors (TLRs) 3, 7, and 8 (27). The activation of these PRRs leads to the activation of interferon regulatory factor (IRF) 3, IRF-7, and nuclear factor kappa-light chain-enhancer of activated B cells (NF-κB), which in turn causes the expression of numerous interferons and cytokines. These include the type I and type III interferons (IFN-α, IFN-β, and IFN-λ, respectively) acting as antiviral cytokines, as well as tumor necrosis factor (TNF) (28). Type I interferons induce an antiviral defense and thereby prevent viral spread (29, 30).

However, the mechanisms to apprehend how CS increases the susceptibility to influenza infection in smokers are insufficiently understood at present. The aim of this study therefore was to investigate how CS modifies the antiviral innate immune response by analyzing DC populations in mice and the immune response of human epithelial cells.

## Methods

### Animal Husbandry

Six- to eight-week-old female C57BL/6J mice were purchased from Charles River Laboratories (Sulzfeld, Germany). Mice were housed in individual ventilated cages under specific-pathogen-free conditions. Animals had access to rodent chow and water *ad libitum*. The temperature, humidity, and day-night cycle of 12 h of the animal facility were kept constant. All animal experiments were approved by the Ministry of Energy, Agriculture, the Environment, Nature and Digitalization, Schleswig-Holstein, Germany (V 242 – 41093/2016 [82-7/16]).

### Smoke Exposure of Mice

Mice were exposed to CS 1 h per day for 24 consecutive days. For dose finding, animals were exposed either to six cigarettes (3R4F, University of Kentucky, USA) per day (1 puff/min) or to 1 puff/min for 3 days, followed by 24 cigarettes per day (4 puffs/min) for the remaining 21 days (Supplementary Figure 4). Air-exposed animals were used as controls. Animals were placed in a whole-body exposure chamber connected to a cigarette smoking robot (in Expose System, Scireq; flexiWare Version 6.1) that automatically lit the cigarettes. Stability of the CS exposure was monitored by daily measurement of particle density using a MDP Pro device (Casella, Bedford, UK) installed at the exit of the exposure chamber. Moreover, cotinine levels were measured in sera at the end of the experiment using a Mouse/Rat Cotinine ELISA (CALBIOTECH, El Cajon, USA). Daily scoring of mice included recording of the body weight using Entris Analytical Balance (±0.01 g, Sartorius, Germany) before every smoke exposure.

### Poly (I:C) Challenge of Mice

Different doses (0.1, 1, or 10 μg) of poly (I:C) (polyinosinic–polycytidylic acid; Sigma-Aldrich, St. Louis, USA) were dissolved in 40 μl phosphate buffered saline (PBS) and administered intranasally under light inhaled anesthesia (sevoflurane) 1 h after the last CS exposure (day 24). PBS was used as control application.

### Lung Function and Organ Removal

See Supplementary Material.

### Bronchoalveolar Lavage

Lungs were rinsed with 1 ml ice-cold PBS. After centrifugation, the cell pellet was resuspended in 200 μl 1x PBS and counted with a hemocytometer. Cytospins were generated using a cytofuge (Cytospin 2, Thermo Shandon GmbH, Germany) and stained with Diff-Quick Staining Set (Siemens Healthcare Diagnostics, Denmark). Alveolar macrophages, neutrophils, and leucocytes were differentiated based on morphological properties under the microscope (Zeiss Standard 16, Oberkochen, Germany). A minimum of 200 cells were evaluated per slide.

### Single Cell Preparation From Murine Lungs

To remove red blood cells from the lungs, the right heart was perfused with ice cold PBS. The left lung lobe was then minced with a scalpel and incubated in digestion buffer (0.5 mg/ml collagenase from *Clostridium histolyticum* [Sigma-Aldrich, USA] in 1x PBS solution) at 37°C in a water bath for 45 min. After digestion, the lungs were homogenized with a 10 ml syringe with a 20G needle (Braun, Germany) and filtered through a cell sieve (70 μm; Corning, USA).

Residual red blood cells in single cell suspensions from the lung were lysed with Geysch'e lysis buffer (10 mM KHCO_3_ [Merck, Germany], 155 mM NH_4_Cl [Merck, Germany], 100 μM EDTA [Sigma-Aldrich, USA]). All single cell suspensions were counted by hemocytometer (dead cell exclusion by trypan blue [0.05% in NaCl solution]) and adjusted to 1 × 10^7^ cells/ml.

### Flow Cytometric Analysis

Left lung homogenates were analyzed by flow cytometry (LSRII; BD FACSDiva software BD Bioscience, USA). Used antibodies are summarized in Supplementary Table 1. Only single cells and non-autofluorescent cells were included in the primary gate. pDC were identified by major histocompatibility complex class II (MHCII), CD11c, and B220. B220 negative CD11c/MHCII positive cells represented all other DCs. The latter were then subdivided into Ly6C negative cDCs and Ly6C positive inflammatory DCs. The subpopulation of cDCs were defined as CD11b positive or CD103 positive cDCs (Supplementary Figure 3).

### RNA Isolation

See Supplementary Material.

### qRT-PCR

See Supplementary Material.

### Cell Culture

Calu-3 cells were cultured on transwells (Falcon, Amsterdam, Netherlands) of 12-well-plates in Dulbecco's minimal essential medium (DMEM, Biochrom, Berlin, Germany) supplemented with 10% heat-inactivated fetal bovine serum (FBS) and 0.01% Gentamicin (Sigma-Aldrich, Darmstadt, Germany) for 1 week until the cells reached confluency. Calu-3 cells were set to air-liquid interface (ALI) for ~1 h prior to smoke exposure.

Madin-Darby Canine Kidney II cells were purchased from the European Collection of Authenticated Cell Culture (ECACC-Sigma-Aldrich, 00062107, Darmstadt, Germany) and maintained at 37°C, 5% CO_2_ in DMEM (Gibco, 11880-036, Life Technologies, Darmstadt, Germany) supplemented with 1% Penicillin/Streptomycin (Gibco, 15140-122, Life Technologies, Darmstadt, Germany), 2 mM glutamine (Gibco, 25030-024, Life Technologies, Darmstadt, Germany), and 10% heat-inactivated FBS (Sigma-Aldrich, F7524, Darmstadt, Germany).

### Preparation of Human PCLS

Lung tissue was acquired from patients who underwent partial resection due to lung cancer at the Hannover Medical School (MHH, Hannover, Germany). Only tissue from macroscopically and microscopically disease free parts of the lung were used for experiments. Human lung slices with peripheral airways were prepared as described before (31). Briefly, a lung lobe was inflated with 2% agarose/medium solution. After the polymerization, the lung lobe was cut into slabs and PCLS of 8 mm in diameter were cut into 300 μm thin slices. Only tissue slices containing airways with intact full smooth-muscle layers, visible regular cilia beating, and comparable airway size as assessed by light microscopy were used in this study. Tissue slices were cultivated submerged in medium (DMEM/F12 supplemented with 1% Penicillin/Streptomycin) at 37°C, 5% CO_2_ overnight. On the day of exposure, PCLS were placed on the inserts of a 12-well-plate (Corning Incorporated, Kennebunk, USA) containing 500 μL medium (DMEM/F12 supplemented with 1% Penicillin/Streptomycin) on the basolateral side. PCLS set to ALI culture were equilibrated for ~1 h prior to smoke exposure.

### Virus Purification

Madin-Darby Canine Kidney II cells at 90% confluency were inoculated with influenza A/California/04/2009/H1N1/pandemic (in the paper referred to as influenza H1N1) virus (provided by the Francis Crick Institute, WHO, London, UK) at a multiplicity of infection of 0.01. Virus was amplified for 40 h at 35°C, 5% CO_2_ in DMEM supplemented with 1% Penicillin/Streptomycin, 2 mM glutamine, and 1 μg/mL N-tosyl-L-phenylalanine chloromethyl ketone (TPCK)-treated trypsin (Thermo Scientific, Waltham, USA). Virus-containing culture supernatant was harvested, clarified at 3,000 × g for 15 min, and loaded onto a 25% sucrose cushion in GNTE buffer (200 mM glycine, 200 mM NaCl, 20 mM Tris-HCl, 2 mM EDTA, pH 7.4). After ultracentrifugation in Optima L80-XP Series ultracentrifuge equipped with a SW32 Ti rotor (Beckman Coulter, CA) at 4°C, and 32,000 rpm for 4 h, the virus pellet was resuspended in GNTE buffer overnight at 4°C, aliquoted, and frozen at −80°C.

### Viral Titer

The viral titer was determined by Focus Forming Assay as previously described (32). Briefly, confluent Madin-Darby Canine Kidney II cells in flat-bottom 96-well-plate were washed and infected with 30 μL of serially-diluted virus (DMEM supplemented with 1% Penicillin/Streptomycin, 2 mM glutamine, 1 μg/mL TPCK treated trypsin without FBS). Virus was allowed to adsorb to cells for 1 h at 35°C, with gentle shaking every 15 min. Then, the inoculum was replaced with 100 μL of media (DMEM supplemented with 1% Penicillin/Streptomycin, 2 mM glutamine, 1 μg/mL TPCK treated trypsin) and 1% Avicel® PH-101 (Sigma-Aldrich, Munich, Germany) for 24 h. The supernatant was removed, and cells were fixed with 100 μL of 4% paraformaldehyde (Sigma-Aldrich, Munich, Germany) at room temperature for 20 min and washed 3 times in PBS for 4 min. Then, 50 μL of 0.3% H_2_O_2_ and 0.5% Triton X-100 were added in each well and incubated at 37°C under humidified atmosphere of 5% CO_2_ for 30 min. The washing was repeated and the cells were stained with primary antibody (1:2,000) (mouse anti-influenza A nucleoprotein, MCA400, Bio-Rad, Germany) diluted in 5% milk/PBS at 37°C for 1 h. Subsequently the cells were washed with 0.02% Tween 20/PBS 3 times for 4 min, and 50 μL of the secondary antibody (1:4,000) (Goat Anti-Mouse IgG-HRP, 170-6516, Bio-Rad, Germany) diluted in 5% milk/PBS were added and incubated at 37°C for 1 h. Cells were washed again and overlayed with 50 μL of TrueBlue™ substrate (Medac Diagnostika, Wedel, Germany) until dark blue foci appeared. The substrate was removed, and the plate was rinsed with tap water and air dried. The foci were manually counted and averaged between the triplicates and expressed as focus forming units (ffu) per mL.

### CS Exposure of Calu-3 and PCLS

The CS exposure with Marlboro red cigarettes (Philip Morris International, Gräfelfing, Germany) was performed using the P.R.I.T.® ExpoCube® system (Fraunhofer ITEM, Hannover, Germany), connected to a cigarette smoking robot (Scireq, Montreal, Canada). Cigarette exposure was controlled by flexiWare software Version 6.1 and monitored for particle mass, CO, CO_2_, and O_2_ via photometer and CO-monitors, respectively, with the DASYLab software (Version 13, National Instruments, Munich, Germany). Calu-3 cells or PCLS were exposed to low dose (two cigarettes) and high dose CS (three cigarettes) with 9 puffs per cigarette within 9 min. The CS exposure of cells and PCLS was repeated 1.5 h later as described above and post-incubated at cell culture condition (37°C and 5% CO_2_) for 16 h after the smoke exposure and prior to infection. Protocol for *in vitro* and *ex vivo* smoking procedure was based on the ISO norm as described previously (33).

### Infection of Calu-3 or PCLS With Influenza H1N1

The cells were inoculated with 5,000 ffu/well in 100 μL and PCLS with 25,000 ffu/well in 250 μL of influenza H1N1. For Calu-3 cells, the inoculum was supplemented with 1 μg/mL TPCK-treated trypsin. Calu-3 cells were infected for 2 h, PCLS at the apical side for 1 h. The cells and PCLS were incubated at 35°C and rocked every 15 min during inoculation to enable homogenous virus infection. Afterwards, the inoculum was discarded, and the cells as well as PCLS were washed two times with 500 μL PBS. After incubation for 48 h, the cells were submerged again. The transepithelial electrical resistance (TEER) measurement was performed as described below and then the apical supernatant was collected for virus detection, LDH release, and apical cytokine quantification. Basolateral medium was also collected for the cytokine measurement. Samples for the cytokine measurements were supplemented with 0.2% protease inhibitor cocktail (P1860, Sigma-Aldrich, Munich, Germany) and stored at −80°C until analysis.

### Transepithelial Electrical Resistance (TEER) Measurements

The TEER of the Calu-3 cells was measured using an EVOM2™ Epithelial Voltohmmeter (World Precision Instruments, Friedberg). Therefore, ALI cultured Calu-3 were submerged with 500 μL of pre-warmed medium on the apical side, incubated at cell culture conditions for 30 min, and TEER was measured between the apical and basolateral compartment. A membrane without cells was used as a blank TEER-value. Final TEER after blank subtraction was expressed as ΔTEER Ω/cm^2^.

### LDH Assay

LDH release assay was performed according to the manufacturer's instructions using Pierce™ LDH Cytotoxicity Assay Kit obtained from Roche (Mannheim, Germany). Thus, 50 μl of apical culture supernatant was incubated with 50 μl of reagent mix (diluted 1:46) in duplicates at RT in the dark for 20 min. The absorbance was measured at 492 nm using a microplate reader infinite F200Pro (Tecan, Männedorf, Switzerland). The reference absorption at 630 nm was subtracted from 492 nm.

### Cytokine Measurement

Cytokines were measured using a multiplex panel (IFN-α2a, IFN-λ, IFN-γ, IL-6, IL-8, monocyte chemoattractant protein 1 [MCP-1], interferon-inducible T cell alpha chemoattractant [I-TAC], IL-15) (MSD, MesoScale Discovery, Gaithersburg, USA) assay. The MSD assay was performed following the manufacturer's instructions using an MSD Sector Imager 2400. Cytokine concentrations were calculated using the Discovery Workbench software (version 4.0, Mesoscale Discovery, Gaithersburg, MD, USA) and based on a 4-fold serial diluted standard. CXCL10/IP-10 was quantified using a commercially available ELISA (R&D Systems, Wiesbaden, Germany) according to the manufacturer's instruction.

### Immunofluorescence Staining and Imaging of Calu-3 Cells

Paraformaldehyde-fixated Calu-3 cells were washed with PBS and permeabilized with 0.3% Triton X-100. Whole filter cultures with cells were blocked with 4% donkey serum in PBS for 30 min and incubated with primary antibody (anti-pan cytokeratin antibody, abcam, Cambridge, UK) diluted at 4°C in 4% donkey serum overnight. Cells were washed three times and incubated with the secondary antibody conjugated to Cy2 was diluted 1:200 at room temperature in 4% donkey serum for 2 h. DAPI was diluted 1:400 in PBS and cells were stained at room temperature for 45 min. Cell as tissue slices were embedded with ibidi mounting medium and stored at 4°C prior to imaging.

Images were acquired using a confocal microscope LSM 800 (Carl Zeiss, Jena, Germany) with x20 objectives. Z-stacks of 20 μm thickness were imaged with image resolution of 2,048 × 2,048.

### Statistical Analyses

Flow cytometry data were analyzed by two-way ANOVA with Tukey's *post-hoc* test followed by paired two-sided *t*-test if Tukey's *post-hoc* test revealed differences among groups. Statistical significance was accepted with *p* < 0.05. All data were analyzed using Graph Pad Prism version 6.0 (GraphPad Prism v6, GraphPad Software Inc., San Diego, USA).

Statistical differences for experiments with Calu-3 cells and PCLS were analyzed by two-way ANOVA with Tukey's *post-hoc* test using GraphPad Prism 8.0.1 (GraphPad Prism v8, GraphPad Software Inc., San Diego, USA).

## Results

### Definition of Smoking Dose Mimicking Moderate to Heavy Smoking

**Figure 1:**
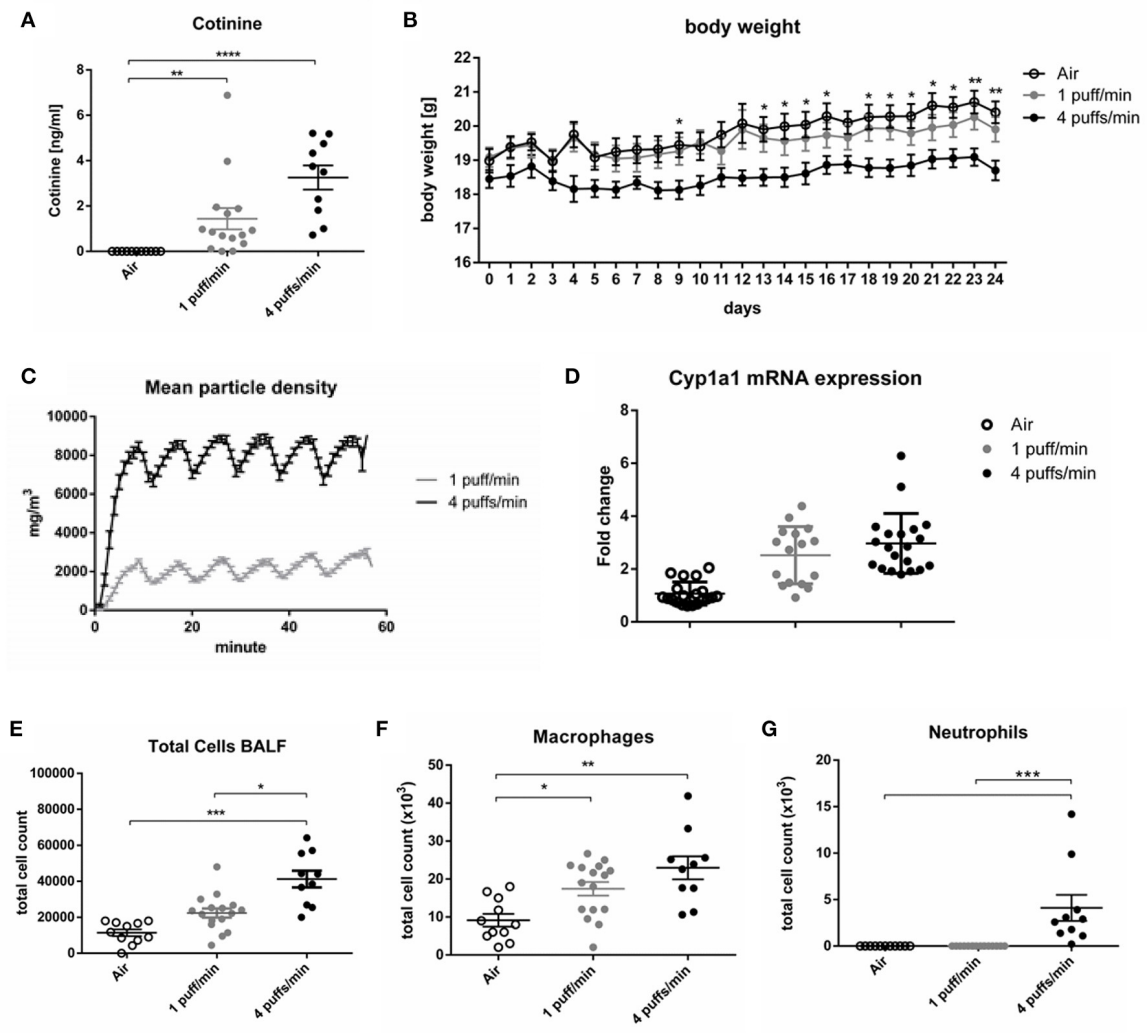
Dose finding for smoking in mice. **(A)** Dose-dependent elevated serum levels of cotinine 24 h after the last CS exposure. **(B)** The body weight gain of female mice over 24 days, *Air vs. 4 puffs/min. **(C)** Mean particle density during the duration of smoke exposure. **(D)** Fold change of the Cyp1a1 mRNA expression in the lung. **(E)** Total cell count in BALF. **(F)** Numbers of macrophages in BALF. **(G)** Numbers of neutrophils in BALF. All data are expressed as mean ± SEM. **(A,E–G)** one-way ANOVA. **(B)** two-way ANOVA. **(A,G)** Dunn's multiple comparison test. **(B,E,F)** Tukey's multiple comparison test.; room air (Air), bronchoalveolar lavage fluid (BALF). *p < 0.05, **p < 0.01, ***p < 0.001, ****p < 0.0001. *n* = 10–16 animals per group in three independent experiments.

As a first step, a dose of cigarette smoking mimicking a moderate-to-heavy smoker, as evidenced by airway inflammation and cotinine levels and without inducing lung structural changes or an overly strong body weight loss, was established. To this end, mice were either exposed to 1 puff/min or 4 puffs/min yielding total particle concentrations of 2.15 ± 0.33 g/m^3^ (1 puff/min) and 7.69 ± 0.76 g/m^3^ (4 puffs/min) in the exposure chamber (Figure 1C). Inhalation of CS by the animals was further confirmed by increased serum cotinine levels after the last cigarette exposure. Cotinine is a more stable metabolite of nicotine. Animals exposed to 1 and 4 puffs/min of CS had significantly higher cotinine concentrations compared to the air-exposed control group. Animals exposed to 4 puffs/min tended to have higher concentrations of the metabolite (3.26 ng/ml ± 0.53) in comparison to 1 puff/min-exposed animals (1.44 ng/ml ± 0.47) (Figure 1A). A slight, non-significant increase in the Cyp1a1 mRNA expression were found in animals exposed to 1 and 4 puffs/min in comparison to air-exposed animals (Figure 1D). Four puffs/min led to a significantly lower body weight gain over time (18.70 ± 0.27 g; day 24) compared to air controls (20.40 ± 0.30 g; day 24). In contrast, 1 puff/min resulted in an only slightly lower weight gain (19.91 ± 0.35 g; day 24) compared to air controls (Figure 1B).

Total cell counts and numbers of macrophages in bronchoalveolar fluid (BALF) increased in a dose dependent manner in both cigarette exposure groups (Figures 1E,F). In contrast, an influx of neutrophils into the alveolar space was only observable in animals exposed to 4 puffs/min (Figure 1G). As expected, lung function measurements including provocation with the bronchoconstrictor methacholine did not differ from air controls as the time of CS exposure was too low to induce airway hyperreactivity (Supplementary Figures 1A–D). Along that line, histology did not show structural changes of the lung architecture (Supplementary Figures 2A,B).

Based on the differences seen in cotinine levels, body weight, and cellular influx into the alveolar space, 4 puffs/min was therefore chosen as smoking dose for all experiments with mice.

### TLR3-Stimulation Increases Lung Inflammatory and Conventional DCs in Smoke-Exposed Mice

**Figure 2:**
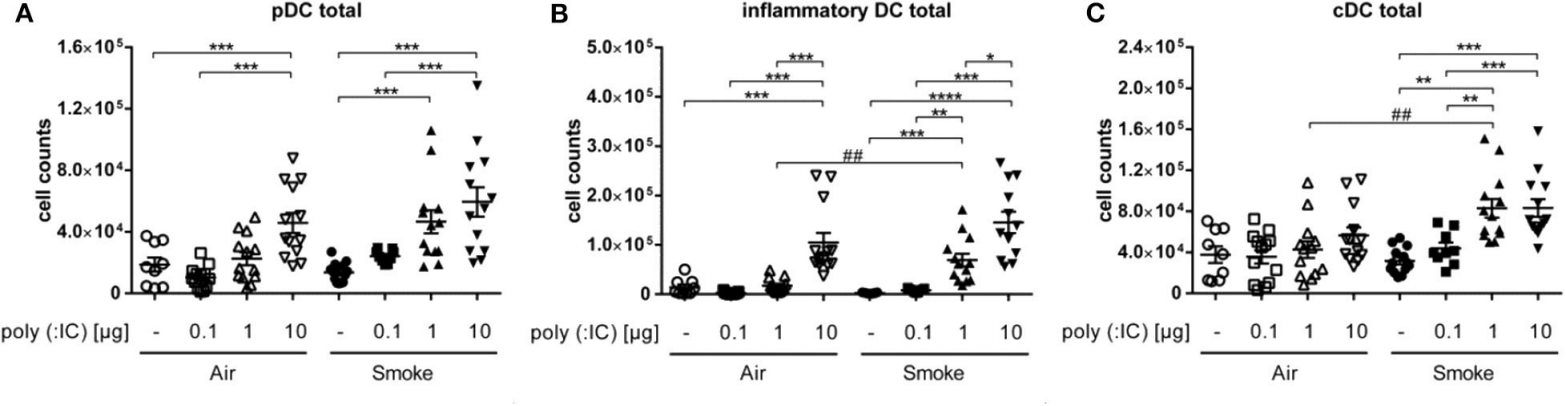
One μg poly (I:C) in smoke-exposed mice induced an increase of DC populations. Cell counts of **(A)** plasmacytoid dendritic cells, **(B)** inflammatory dendritic cells, and **(C)** conventional dendritic cells from single cell suspension of lungs. Empty symbols show air-exposed and filled symbols smoke-exposed animals. Every symbol represents one animal. Individual values and mean, Comparison of different poly (I:C) doses each within CS- or air-exposed animals *p ≤ 0.05, **p ≤ 0.01, ***p ≤ 0.001, ****p ≤ 0.0001; Comparison of identical poly (I:C) dose between CS- and air-exposed animals ^*##*^*p* ≤ 0.01, paired two-sided *t*-test. *n* = 9–13 animals per group in three independent experiments.

To investigate how a viral stimulus alters subpopulations of lung DCs in CS-exposed mice, we performed flow cytometry analysis of whole lung single cell suspensions. pDCs (CD11c^high^, MHCII^high^, B220^high^) are considered to be important players in antiviral immunity as they can rapidly secrete type 1 interferons. Sole exposure to smoke did not significantly change numbers of pDCs compared to the air group. The intranasal administration of poly (I:C) led to a dose-dependent increase in the number of pDCs, which was not further increased by smoke (Figure 2A).

As with pDCs, the numbers of iDCs (CD11c^high^, MHCII^high^, B220^low^, Ly6C^high^) showed no difference between the exclusively smoke- vs. air-exposed animals. Similar to pDC, poly (I:C) alone led to a dose dependent increase in iDCs. However, in smoke-exposed mice, 1 μg poly (I:C) led to significantly higher number of the inflammatory cells as compared to air controls that received the same dose (Figure 2B).

Interestingly, unlike pDCs and iDCs, numbers of cDCs (CD11c^high^, MHCII^high^, B220^low^, Ly6C^low^) seemed to be unaffected by the administration of poly (I:C) in the air group. However, addition of the viral mimetic to smoke-exposed animals increased the numbers of the cells at doses of 1 as well as 10 μg in comparison to the only smoke and the corresponding poly (I:C)-treated air controls (Figure 2C), which was significant at 1 μg only.

**Figure 3:**
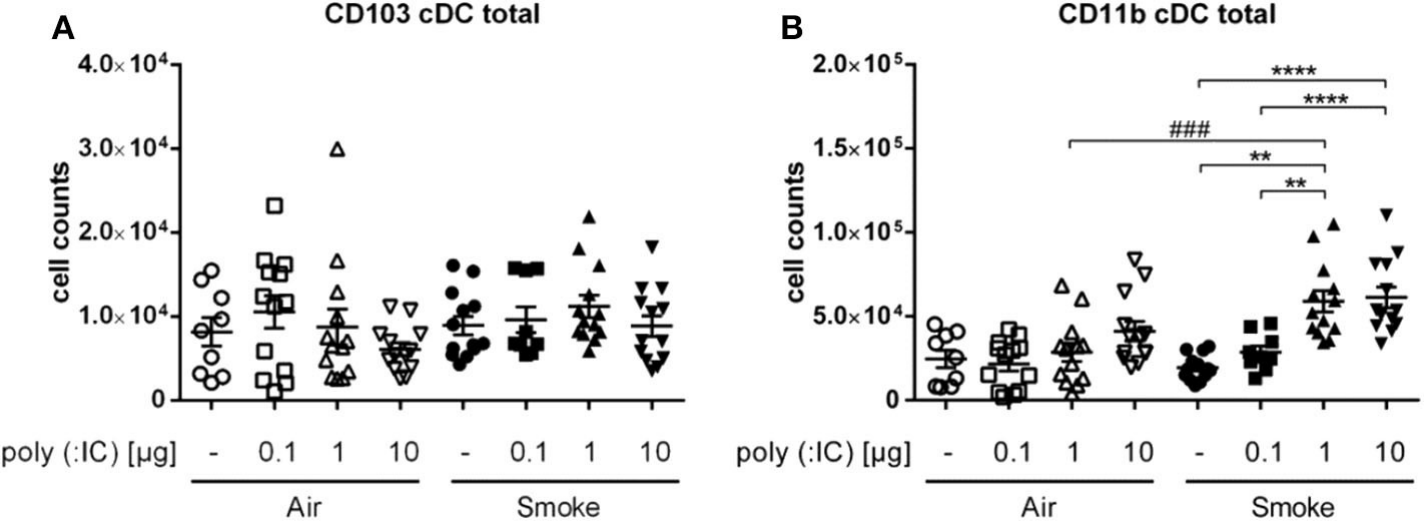
CS and poly (I:C) affected only CD11b^+^ of cDC subpopulations. Results show cell counts of **(A)** CD103^+^ cDCs and **(B)** CD11b^+^ cDCs from single cell suspension of lungs. Empty symbols show air-exposed and filled symbols smoke-exposed animals. Comparison of different poly (I:C) doses each within CS- or air-exposed animals **p ≤ 0.01, ****p ≤ 0.0001; Comparison of identical poly (I:C) dose between CS- and air-exposed animals ^*###*^*p* ≤ 0.001, paired two-sided *t*-test. *n* = 9–13 animals per group in three independent experiments.

To assess which subpopulation of cDC could be responsible for this difference, we investigated CD11b^+^ and CD103^+^ DCs. While the latter remained unchanged, CD11b^+^ cDCs showed a dose dependent increase in response to poly (I:C). Especially in the smoke-exposed group of animals, the increase was significantly higher in the animals exposed to smoke with the 1 μg poly (I: C) dose (5. 9*10^4^ cells ± 6.3*10^4^) compared to the corresponsding air group. Thus, the differences seen for total cDC were confined to the CD11b^+^ cDC subpopulation (Figure 3).

Taken together, smoke exposure alone did not affect DC subsets in the lungs, whereas subsequent exposure to the TLR-3 ligand poly (I:C) increased numbers of pDC, iDC, and cDC in the lung.

### Both CS Exposure and Influenza H1N1 Infection Impaired Barrier Integrity of Calu-3 Cells but Not of Lung Tissue Slices

Intercellular communication between dendritic and epithelial cell types is essential for inducing an adapted antiviral immune response. We hypothesized that the alterations seen in DCs could result from altered signaling by the epithelial barrier. To therefore evaluate the impact of acute CS exposure on epithelial cell barrier function as well as the local immune response to subsequent virus infection in the human lung, we compared the virus load, TEER, and cytokine release upon influenza H1N1 infection of Calu-3 cells and lung slices after air or CS exposure.

**Figure 4:**
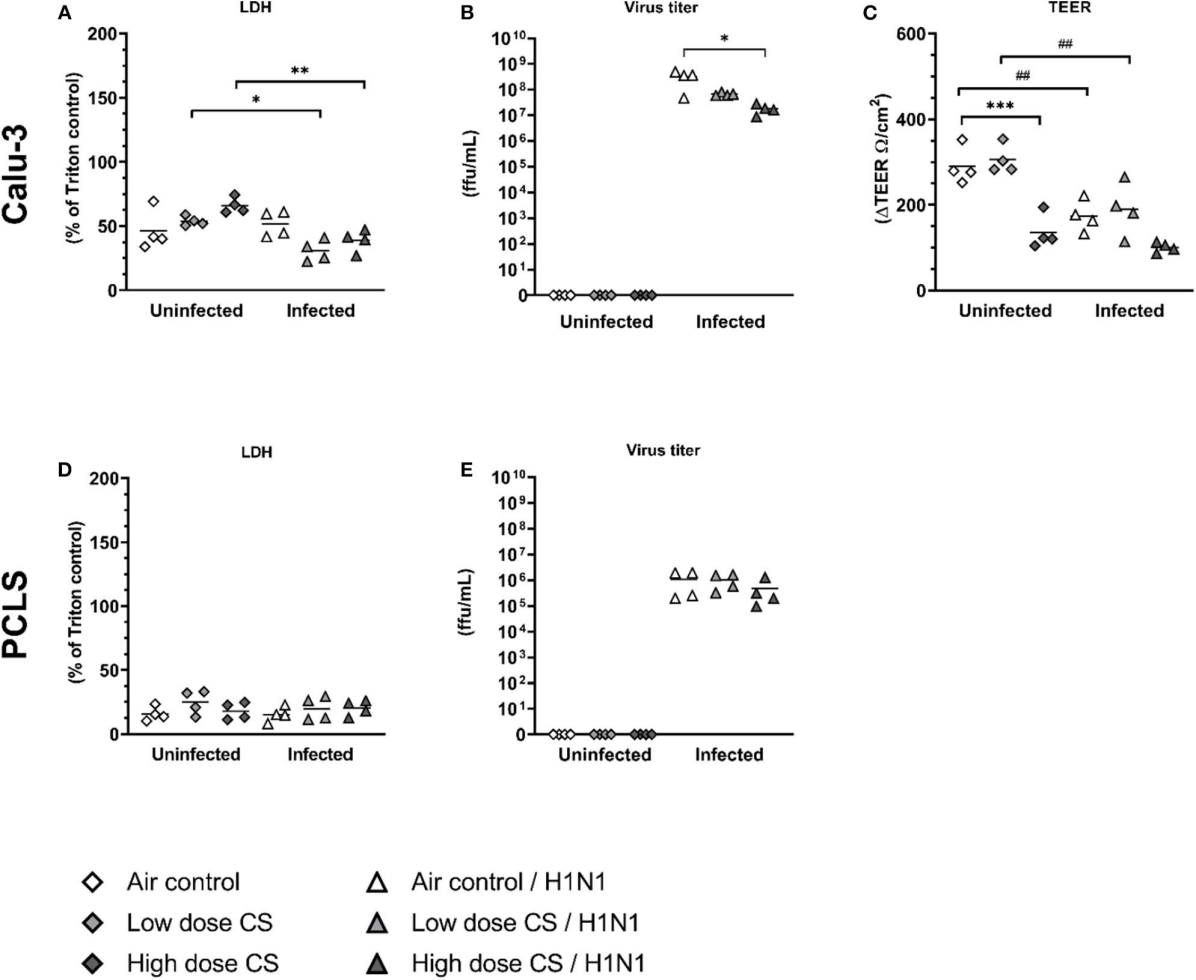
**(A–E)** CS decreased viral replication in influenza H1N1-infected Calu-3 cells, whereas it was unchanged in PCLS. Calu-3 cells were exposed twice to either air or two doses of CS at the ALI. Following exposure, the cells were post-incubated for 24 h and then infected with influenza H1N1 (5,000 ffu/well for Calu-3 and 25,000 ffu/well for PCLS) at the apical surface. Reduction of TEER was observed after high dose CS or influenza H1N1 infection in exposed Calu-3 cells 48 h post infection. Virus titer was reduced in CS-exposed Calu-3 cells but not in CS-exposed PCLS. LDH release was reduced in CS-exposed and influenza H1N1-infected Calu-3 cells but not in PCLS. Every symbol represents an independent experiment (and donor for human PCLS). *n* = 4 independent experiments with four technical replicates each. *p < 0.05, **p < 0.01, ***p < 0.001 compared with respective air control within uninfected/infected group analyzed by Tukey's multiple comparisons test, ^*##*^*p* < 0.01 uninfected vs. infected in air or CS exposed cells, analyzed by Tukey's multiple comparisons test.

**Figure 5:**
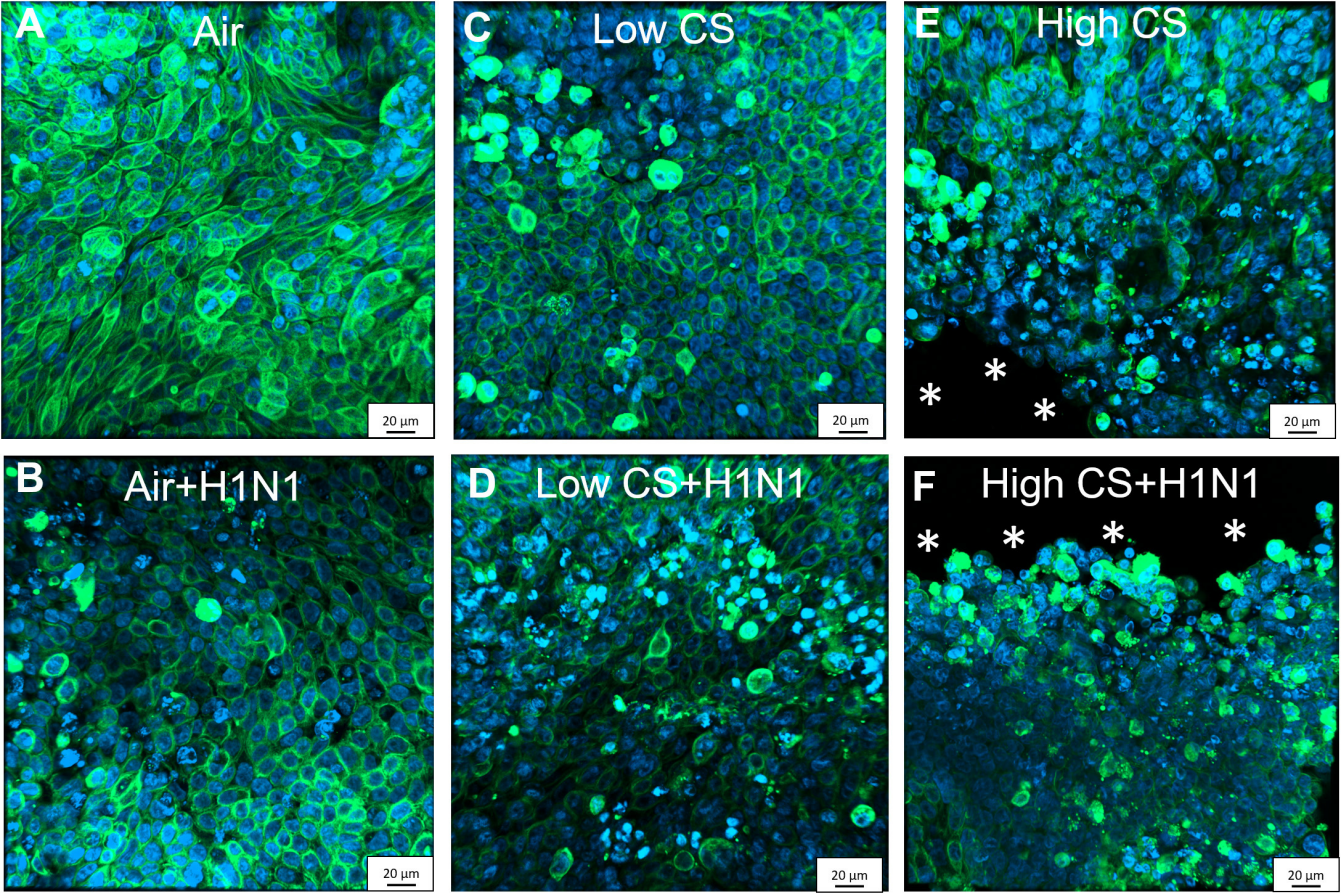
CS exposure induced morphological changes in confluent Calu-3 cells due to cytotoxicity, which were enhanced by cytopatic effects of influenza H1N1 infection. Calu-3 cells were exposed to either air or two doses of CS at the ALI. Following exposure, the cells were post-incubated for 24 h and then infected with influenza H1N1 (5,000 ffu/well) at the apical surface. The inoculum was removed and cells were post-incubated for 48 h prior to the staining. Fixated Calu-3 cells were stained for pan cytokeratin (green) and cell nuclei with DAPI (blue) after **(A)** air only, **(B)** low dose CS, **(C)** high-dose CS, **(D)** air + influenza H1N1, **(E)** low dose CS + influenza H1N1, and **(F)** high-dose CS + influenza H1N1. Asterisk (*) shows cell detachment.

CS exposure alone showed a dose-dependent cytotoxicity on Calu-3 cells as measured by LDH release, which was not statistically significant (Figure 4A). Influenza H1N1 infection of air-exposed Calu-3 cells did not lead to increased cytopathic effect as assessed by LDH release, whereas CS exposure prior to influenza H1N1 infection resulted in a lower release of LDH compared to uninfected and exposed cells. Virus titers remained comparable in the low dose CS-exposed cells compared to air-exposed, but decreased in high dose CS-exposed cells after H1N1 infection (Figure 4B). As the result of reduced LDH release as well as reduced viral load was unexpected, we further evaluated how the CS exposure affects the epithelial barrier function. High dose CS exposure significantly impaired barrier function as shown by decreased TEER value, while low dose CS was well-tolerated and did not impact the TEER value (Figure 4C). Influenza H1N1 infection of air-exposed cells reduced epithelial barrier integrity. The impaired barrier function was also observed in low dose CS-exposed and influenza H1N1-infected cells. At high dose CS, epithelial integrity was already impaired, which was not further augmented by influenza H1N1 infection. To then evaluate why LDH release as well as virus titer decreased in high dose CS-exposed and infected cells, immunofluorescence staining of Calu-3 cells was performed (Figure 5).

In PCLS, CS exposure did not increase LDH activity at low or high dose of CS (Figure 4D). Influenza H1N1 infection showed no cytopathic effect in air-exposed or CS-exposed and infected cells (Figure 4E), and replication remained unaffected in lung tissue slices *ex vivo*. TEER measurement could not be implemented for lung tissue slices.

To analyze why the influenza titer decreased at high CS dose in Calu-3 cells, the cells were stained for pan-cytokeratin and cell nuclei. Air-exposed control confirmed cell confluency 72 h post exposure (Figure 5A). Influenza H1N1 infection showed first cytopathic effect in cell layer (Figure 5D). Low dose CS slightly induced cytotoxicity (Figure 5B), which was further increased in influenza H1N1-infected cells (Figure 5E). High dose CS induced partial detachment of cell layer (marked with asterisks) in exposed cells (Figure 5C), which was even stronger in high dose CS-exposed and influenza H1N1-infected cells (Figure 5F). This is in line with data shown in Figure 4 as a lower number of Calu-3 cells led to reduction of total influenza H1N1 titer in culture supernatant and confirmed lower LDH release. These data indicate that selected high dose CS was cytotoxic for the Calu-3 cells and low dose CS showed how CS affects epithelial response upon influenza H1N1 infection.

### Acute CS Exposure Decreased Release of Antiviral Cytokines to Influenza H1N1

Next, we aimed to analyze whether acute CS exposure affected the host response by analyzing cytokines and chemokines for innate and adaptive immune response against influenza H1N1 in epithelial cells (Calu-3) and PCLS as a more complex, immunocompetent lung tissue.

**Figure 6:**
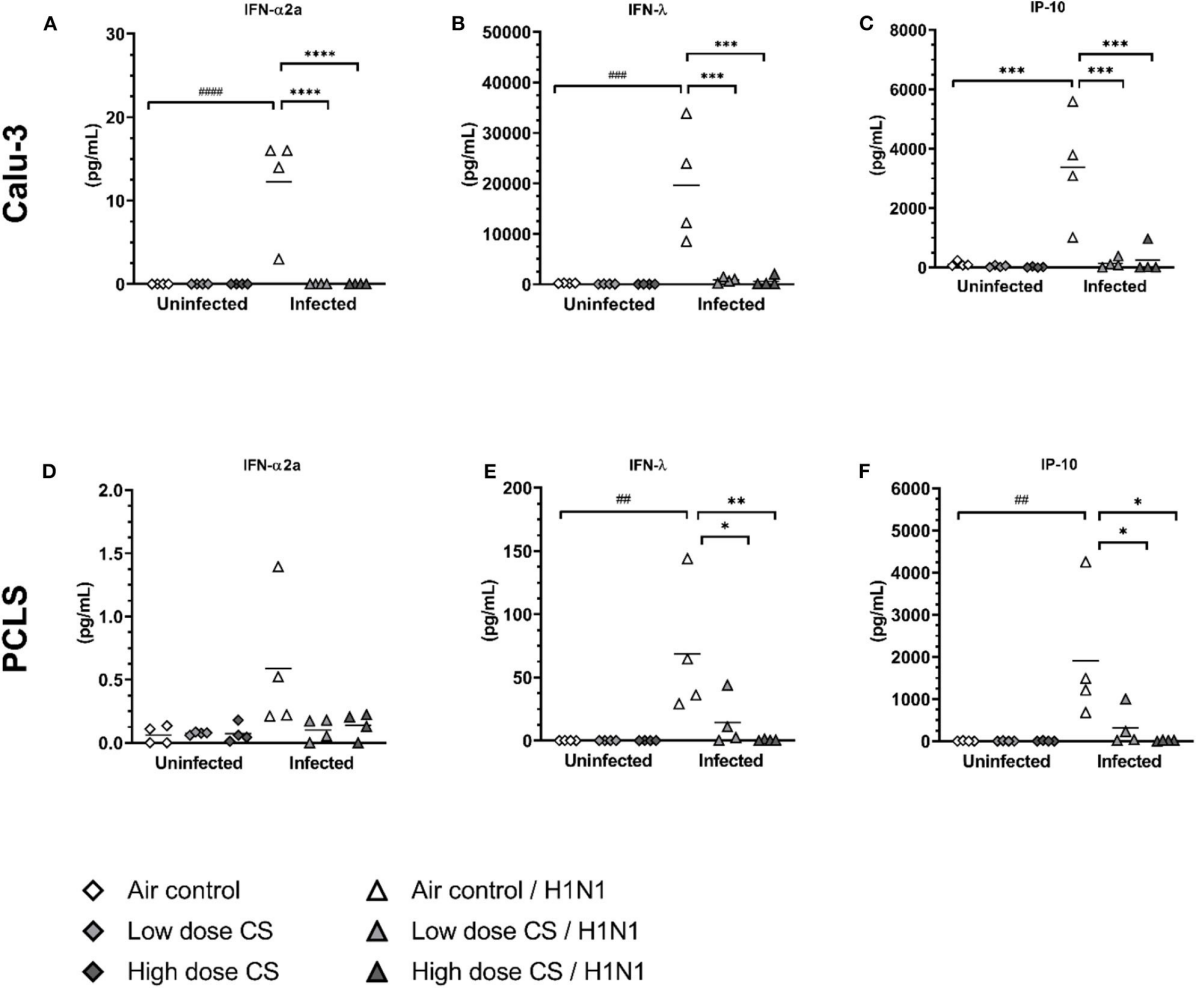
**(A–F)** CS impaired antiviral cytokine release upon H1N1 infection in Calu-3 cells and human PCLS. Calu-3 cells and human PCLS were exposed to either air or two doses of CS at the ALI. Following exposure, the cells/PCLS were post-incubated for 24 h and then infected with influenza H1N1 (5,000 ffu/well for Calu-3 and 25,000 ffu/well for PCLS) at the apical surface. Antiviral cytokines were induced in air-exposed and influenza H1N1-infected cells/PCLS but were inhibited in CS-exposed cells/PCLS 48 h post infection. Every symbol represents an independent experiment (and donor for human PCLS). *n* = 4 with four technical replicates, *p < 0.05, **p < 0.01, ***p < 0.001, ****p < 0.0001 compared with respective air control within uninfected/infected group analyzed by Tukey's multiple comparisons test, ^*##*^*p* < 0.01, ^*###*^*p* < 0.001, ^*####*^*p* < 0.0001 air control vs. air infected control, analyzed by Tukey's multiple comparisons test.

Secretion of antiviral cytokines to induce an antiviral state in uninfected epithelial cells is crucial to prevent viral spread. We observed that CS exposure itself had no effect on the baseline level of IFN-α2a, IFN-λ, and IP-10 both at low and high CS doses in Calu-3 cells or PCLS (Figures 6A–C). As expected, infection of air-exposed Calu-3 cells with influenza H1N1 significantly induced the release of IFN-α2a (0 vs. 12.3 pg/mL), IFN-λ (220 vs. 19,647 pg/mL), and IP-10 (119 vs. 3,370 pg/mL) compared to uninfected controls. Strikingly, previous CS exposure of Calu-3 cells led to a complete suppression of antiviral cytokine release upon H1N1 infection already at the low dose.

Similar to Calu-3 cells, air-exposed PCLS infected with influenza H1N1 resulted in increased release of IFN-λ (0.1 vs. 68 pg/mL) and IP-10 (4 vs. 1,911 pg/mL) compared to uninfected air-exposed control (Figures 6E,F). Minor increase of IFN-α2a release upon infection was not statistically significant (Figure 6D). CS exposure of PCLS dose-dependently prevented the antiviral response to influenza H1N1 infection. This resulted in reduced levels of IFN-λ (68 vs. 14 pg/mL at low dose CS or 68 vs. 0.5 pg/mL at high dose CS) as well as IP-10 response (1,911 vs. 323 pg/mL at low dose CS or 1,911 vs. 18 pg/mL at high dose CS). The immune response was due to active virus replication as UV-inactivated control showed no significant increase in cytokine secretion (Supplementary Figure 5).

Overall, acute CS exposure prevented the induction of the antiviral response of epithelial cell culture of Calu-3 cells and in complex lung tissue slices (PCLS) to influenza H1N1.

### CS Impaired Pro-inflammatory Immune Response to Influenza H1N1

**Figure 7:**
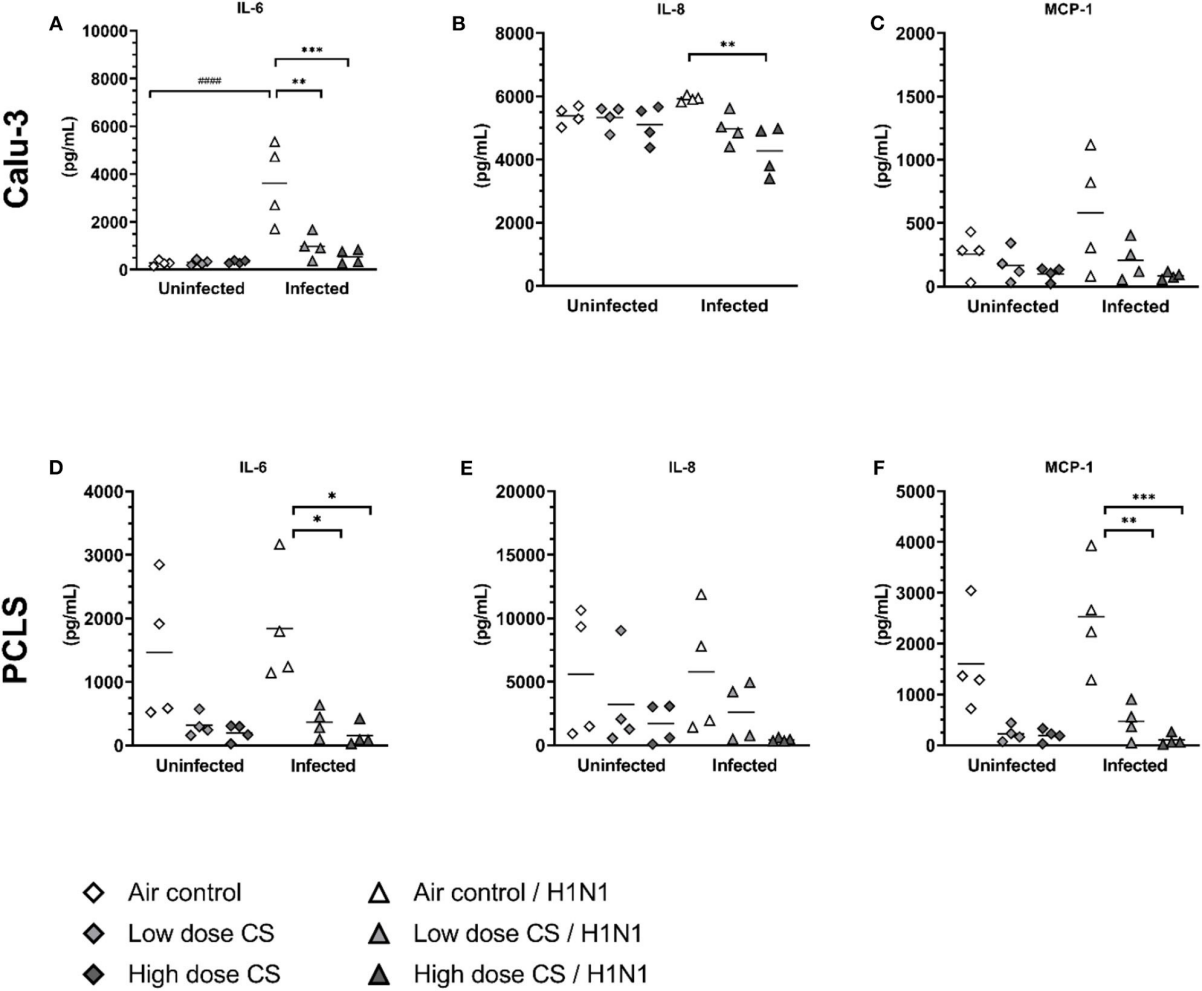
**(A–F)** CS impaired pro-inflammatory immune response in influenza H1N1-infected Calu-3 cells and PCLS. Calu-3 cells and human PCLS were exposed to either air or two doses of CS at the ALI. Following exposure, the cells/PCLS were post-incubated for 24 h and then infected with influenza H1N1 (5,000 ffu/well for Calu-3 and 25,000 ffu/well for PCLS) at the apical surface. Pro-inflammatory cytokines were induced in air-exposed and influenza H1N1-infected cells/PCLS but were inhibited in CS-exposed cells/PCLS 48 h post infection. Every symbol represents an independent experiment (and donor for human PCLS). *n* = 4 with four technical replicates each, *p < 0.05, **p < 0.01, ***p < 0.001 compared with respective air control within uninfected/infected group, ^*###*^*p* < 0.001 air control vs. air infected control, analyzed by Tukey's multiple comparisons test.

CS exposure of Calu-3 cells had no effect on baseline levels of the pro-inflammatory cytokines IL-6 (Figure 7A). In PCLS, CS induces an antiinflammatory effect resulting in reduced levels of IL-6 (1,467 vs. 316 pg/mL at low dose CS or 1,467 vs. 198 pg/mL at high dose CS), although due to high variability this reduction was not statistically significant (Figure 7D). Influenza H1N1 infection of air-exposed Calu-3 cells significantly induced IL-6 (277 vs. 3,623 pg/mL) compared to uninfected air-exposed control. This induction was significantly suppressed in CS-exposed Calu-3 cells (983 pg/mL in low dose CS group and at 550 pg/mL in high dose CS) upon influenza H1N1 infection. Similarly, acute CS-exposure impaired pro-inflammatory immune response to influenza H1N1 in PCLS by reduced levels of IL-6 (1,837 vs. 364 pg/mL at low dose CS or 1,837 vs. 157 pg/mL at high dose CS).

CS exposure of Calu-3 cells had no effect on baseline levels of IL-8 (Figure 7B), however in PCLS tendency toward reduced levels of IL-8 resulted in 5,588 vs. 3,226 pg/mL at low dose CS or 5,588 vs. 1,685 pg/mL at high dose CS (Figure 7E). Influenza H1N1 infection of Calu-3 cells as well as PCLS did not lead to IL-8 induction. An anti-inflammatory effect of CS was observed on IL-8 levels in influenza H1N1 group. In CS-exposed and infected Calu-3 cells the release of IL-8 was reduced from 5,588 to 3,226 pg/mL at low dose CS and from 5,588 to 1,685 pg/mL at high dose CS. In the same line, IL-8 release was reduced in acute CS-exposed and influenza H1N1 PCLS (5,760 vs. 2,600 pg/mL at low dose CS or 5,760 vs. 441 pg/mL at high dose CS). Although this effect did not reach statistical significance, IL-8 levels were reduced in every donor.

The effect of CS exposure on MCP-1 release was even more prominent. Minor reduction in MCP-1 release was observed in CS-exposed Calu-3 cells (Figure 7C) but MCP-1 release in CS-exposed PCLS decreased from 1,603 to 226 pg/mL at low dose CS or from 1,603 to 192 pg/mL at high dose CS (Figure 7F). Again, this reduction was not significant due to donor variability, but MCP-1 levels were reduced in every donor. Influenza H1N1 infection of air-exposed cells as well as PCLS led only to minor increase in MCP-1 release. CS exposure prior to influenza H1N1 infection dose-dependently reduced MCP-1 release in Calu-3 cells. Similarly in PCLS, acute CS exposure significantly reduced MCP-1 levels from 2,529 to 469 pg/mL at low dose CS and from 2,529 to 99 pg/mL at high dose CS compared to air-exposed influenza H1N1-infected PCLS.

TNF-α could not be quantified in Calu-3 cells as it was under detection limit (data not shown). In PCLS CS exposure did not reduce TNF-α release compared to air exposed control. H1N1 infection of air exposed PCLS induced TNF-α (0.4 vs. 2.4 pg/mL) compared to uninfected air exposed control. This induction was impaired in cigarette smoke exposed PCLS by reduced release of TNF-α at 1.2 pg/mL in low dose CS group and at 1.1 pg/mL in high dose CS upon influenza H1N1 infection, although it did not reach statistical significance (Supplementary Figure 6).

### CS Impaired T Cell Response to Influenza H1N1

Orchestrated by DCs, the activation of antigen-specific T cells as well as natural killer (NK)-cells is important in protecting the organism against virus infection. To determine the impact of smoke exposure on subsequent T cell response, we evaluated induction of cytokines acting as biomarker for T cell response, namely IFN-γ, IL-15, and I-TAC. These cytokines are secreted upon activation (IFN-γ) or they regulate activation and proliferation of T cells/NK cells and act chemotactically for activated cells (I-TAC). Moreover, they control responsiveness of monocytes and DC.

**Figure 8:**
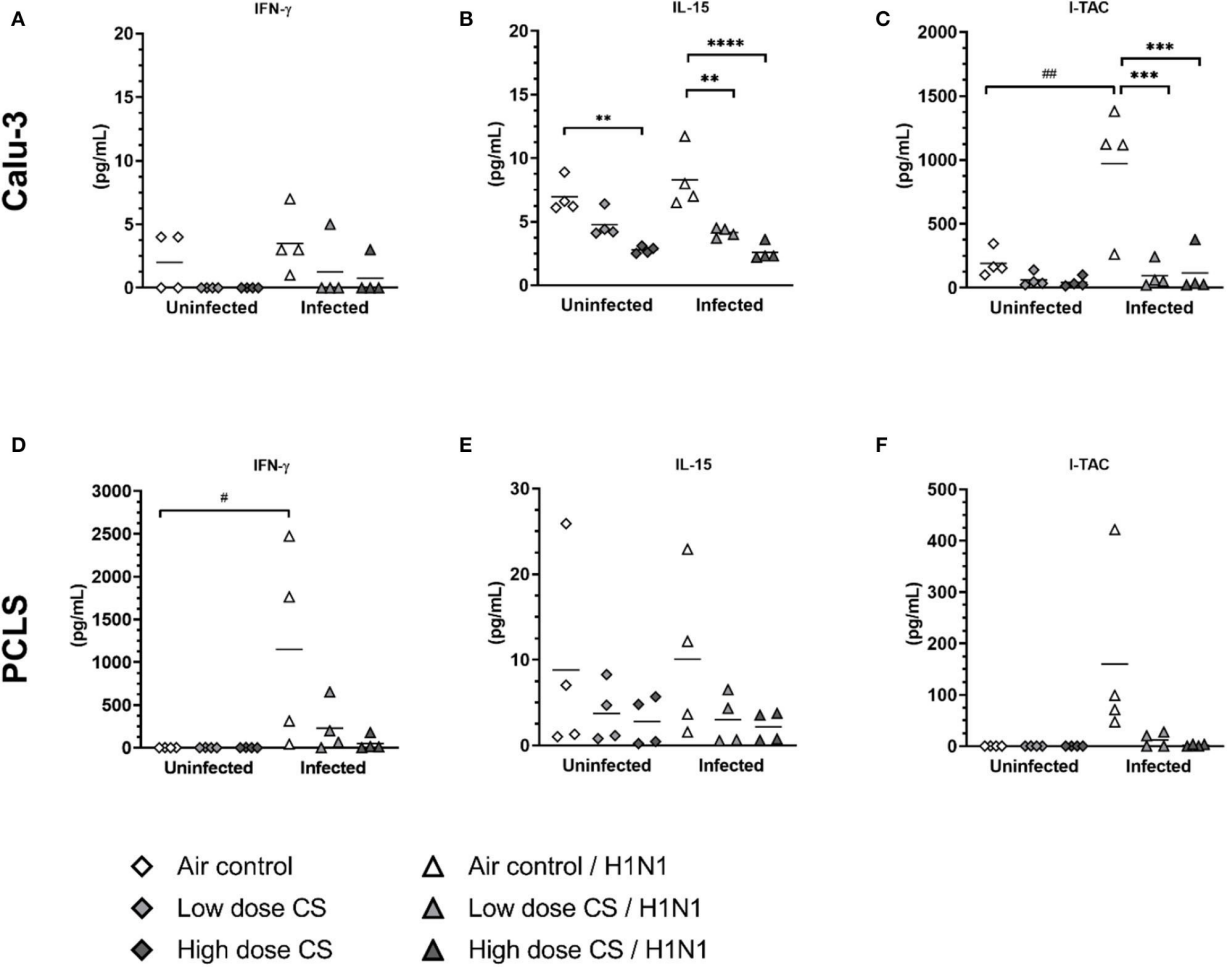
**(A–F)** CS impaired T cell activating response to influenza H1N1-infected Calu-3 cells and PCLS. ALI cultured Calu-3 cells and human PCLS were exposed to either air or CS (at low and high dose). Following exposure, the cells/PCLS were post-incubated for 24 h and then infected with influenza H1N1 (5,000 ffu/well for Calu-3 and 25,000 ffu/well for PCLS) at the apical surface. T cell activating cytokines were induced in air-exposed and influenza H1N1-infected cells/PCLS but were inhibited in CS-exposed cells/PCLS 48 h post infection. Every symbol represents an independent experiment (and donor for human PCLS). *n* = 4 with four technical replicates each, *p < 0.05, **p < 0.01, ***p < 0.001, ****p < 0.0001 compared with respective air control within uninfected/infected group, ^#^*p* < 0.05, ^*##*^*p* < 0.01 air control vs. air infected control, analyzed by Tukey's multiple comparisons test.

CS exposure itself had no significant effect on baseline levels of IFN-γ release in Calu-3 cells (Figure 8A) or PCLS (Figure 8D). Influenza H1N1 infection did not induce IFN-γ release in air-exposed Calu-3 cells; however, IFN-γ was strongly induced in air-exposed PCLS with an increased release from 2 to 1,148 pg/mL upon infection. This induction was impaired in previously CS-exposed PCLS resulting in levels of IFN-γ at 229 pg/mL in low dose CS group and at 51 pg/mL in high dose CS upon influenza H1N1 infection.

CS exposure dose-dependently reduced baseline levels of IL-15 in Calu-3 cells (7.0 vs. 4.8 pg/mL at low dose CS or 7.0 vs. 2.8 pg/mL at high dose CS) and showed tendency toward IL-15 inhibition in PCLS compared to air-exposed control (Figures 8B,E). The release of IL-15 was not induced upon influenza H1N1 in Calu-3 cells or PCLS. IL-15 levels remained reduced in CS-exposed and H1N1-infected Calu-3 cells in a dose-dependent manner (4.2 pg/mL in low dose CS group and at 2.6 pg/mL in high dose). These data are comparable to PCLS, where reduced IL-15 levels were detected at both CS concentrations upon influenza H1N1 infection, which were, however, not statistically significant.

The release of chemoattractant I-TAC showed a tendency toward dose-dependent reduction to baseline levels in CS-exposed Calu-3 cells (Figure 8C). Only baseline levels of I-TAC were measured in uninfected PCLS, therefore no effect of CS on I-TAC release could be detected in uninfected lung tissue (Figure 8F). I-TAC release was induced in Calu-3 cells from 191 to 972 pg/mL and in PCLS from 0.1 to 160 pg/mL upon influenza H1N1 infection.

## Discussion

Smokers are more susceptible to influenza virus infections than non-smokers (34), but the mechanisms to apprehend how CS increases the susceptibility to influenza infection are poorly understood. In this study, we demonstrated that prolonged CS exposure of mice lead to increased airway inflammation with a prominent rise of inflammatory DCs upon challenge with the viral mimetic poly (I:C). Furthermore, we investigated *in vitro* whether these changes are linked to smoke and virus-induced innate epithelial responses. However, the results showed the opposite since smoke exposure inhibited both the antiviral and the inflammatory response of human epithelial cells. Similar findings were made *ex vivo* in human lung tissue.

The pathophysiological role of DCs in viral infection and influenza virus in particular has been studied comprehensively in rodents and human cell culture experiments (35–38). DCs form a highly developed network within or below the epithelial layer consisting of different DC subtypes. In this study, we evaluated how CS, followed by stimulation with a TLR3 ligand, affects DC populations *in vivo*. Mouse adapted influenza virus strains like the PR8 live virus are able to infect and replicate in C57BL/6 wild type mice and even showed a increased mortality in combination with CS exposure (39). However, the human pandemic strain H1N1 does infect mice but does not induce an adequate immune response (40). For this reason, we applied the synthetic viral mimetic poly (I:C) to study changes of DC subpopulations in mice. CS increased the total numbers of cells, macrophages, and neutrophils in BAL in subchronically smoke-exposed mice, which is regularly seen in smoke-exposed mice (41, 42). Because DCs have a pivotal role in the regulation of immune responses, we also investigated the influence of CS on DC subsets in the same animals. We found that only high doses of poly (I:C) are able to affect DC population in the lung in air controls. However, CS exposure prior to poly (I:C) challenge seems to affect the responsiveness of DC toward the TLR-ligand. Already low concentrations of poly (I:C) are able to significantly increase the number of pDC, iDC, and cDC in comparison to the only smoke control. Moreover, the direct comparison of air- and smoke-exposed animals revealed that these effects are for cDC and iDC dependent of the CS exposure and that the increase in cDC is mainly due to CD11b^+^ cDC.

An increase of pulmonary DCs in response to CS in mice has been reported previously (43–48) with only few studies observing reduced numbers in CS-exposed and antigen challenged mice (49, 50). In smoke-exposed mice, an early increase in cell counts compared to the air group at the same stimulus with a defined poly (I:C) dose often occurs. This difference is particularly noticeable at a medium dose. The epithelial and dendritic cell cross talk plays an important role in the orchestration of immune responses. Both cells are located in close proximity and build the structural and immunological first line of defense against exogenous invaders. Here, epithelial cells do not only build a physical wall but are also able to sense pathogens, produce anti-microbial mediators, and activate/modulate immunological responses. To evaluate impact of CS on epithelial cells and their responsiveness toward influenza infections, epithelial cells as well as *ex vivo* cultured lung tissue was analyzed. Calu-3 cells, which represent epithelial cells from the proximal airways, and human precision-cut lung slices, which contain alveolar and airway epithelial cells as well as macrophages, DCs, T cells, and other cell types present in the lower respiratory tract, were exposed to CS. Acute CS exposure damaged the epithelial barrier. High doses of CS induced cytotoxicity in Calu-3 cells and thereby contributed to increased permeability. In PCLS no significant loss of viability was observed at low or high dose CS exposure. PCLS do not build an intact barrier between apical and basolateral compartment on transwells, and therefore no increased permeability could be evaluated. These findings are in line with previous studies showing that CS directly increases permeability of epithelial and alveolar cells (51–53). Tatsuta et al. also observed decreased TEER and increased permeability after Calu-3 cells were treated with cigarette smoke extract. They could show that cigarette smoke extract altered gene expression of claudin-1, claudin-3, claudin-4, claudin-7, claudin-15, occludin, E-cadherin, junctional adhesion molecule-A (JAM-A), and zonula occludens-1 (ZO-1) (54). Although we don't see a decreased TEER at low dose CS, but we do at high dose CS, the effect on TEER could be partly due to increased cytotoxicity. The destruction of the airway epithelial cell layer may act as a danger signal for surrounding cells and thereby lead to a prominent release of pro-inflammatory cytokines. Moreover, CS itself contains pro-inflammatory components such as endotoxin, which could induce an inflammatory response. Contrary to this expectation, CS exposure did not change the release of pro-inflammatory cytokines (IL-6, IL-8, MCP-1) in Calu-3 epithelial cells as well as in human PCLS. CS exposure dose-dependently reduced IL-15 release of Calu-3 cells, which plays an important role in immune responses by regulating proliferation, survival, and functions of lymphocytes and NK cells. In PCLS, CS even dose-dependently reduced pro-inflammatory cytokines (IL-6, IL-8, and MCP-1), and similar to Calu-3 cells, the release of IL-15 was inhibited in PCLS as well. Levels of antiviral and other T cell/NK cell regulating cytokine of CS-exposed PCLS were close to baseline. Thus, we observed no increased pro-inflammatory cytokine and chemokine response neither in Calu-3 cells nor in viable lung slices after exposure to CS. Interestingly, that induction of immune response toward infection was also reported controversially in publications. A number of studies reported that secretion of several pro-inflammatory cytokines such as IL-6, IL-8, IP-10, and Rantes was suppressed (55, 56), whereas others reported induced expression of IL-1β and IL-8 in human bronchial epithelial cells (HBE-14o) after CS extract exposure (57). With this in line, CS extract exposure of pDCs from healthy individuals also confirmed impaired release of IL-8, TNF-α, IL-6, and IFN-α (58). Thus, the impaired barrier function of epithelial cells was not accompanied by a prominent pro-inflammatory response, suggesting that airway inflammation observed in mice after CS exposure is not due to an initial pro-inflammatory response of the airway and alveolar tissue. It supports the hypothesis that airway inflammation, as observed in mice after 24 days of exposure to CS, is the result of a rather secondary driven inflammation process during ongoing lung injury.

Our results showed that viral infection after exposure of airway epithelial cells and lung tissue *ex vivo* to CS further decreased viability, increased permeability, and impaired antiviral and pro-inflammatory responses. Influenza H1N1 infection impaired epithelial barrier function in air exposed and low dose cigarette smoke exposed Calu-3 cells without causing cytotoxicity. This is in line with the study from Duffney et al., who showed immunosuppressive effect of CS exposure on IP-10, IL-6, and IL-8 secretion in poly (I:C) stimulated human small airway epithelial cells (56). Wu et al. previously showed that submerged exposure of human PCLS to cigarette smoke extract resulted in suppressed pro-inflammatory and antiviral response to influenza due to oxidative inhibition of RIG-I (59). In addition, the study from Jasper et al. found impaired interferon induction in nasal epithelial cells from smokers after infection with influenza, which was caused by suppression of the key transcription factors such as IFN regulatory factor 7 (60). The study of Wu et al. furthermore suggested that active smoking results in epigenetic modifications leading to downregulation of RIG-I and TLR3 thereby increasing the risk and severity of viral respiratory tract infections in smokers (61). Challenging human smoking volunteers with attenuated influenza virus lead to the suppression of infiltrated γδ T cells (62) and the nasal mucosal response in these volunteers showed reduced IL-6 and increased viral load in smokers (63). Additionally to the published data, this study, using human lung slices as an intact complex lung tissue, highlights the impaired T cell response to the virus and facilitate immune disfunction. Thus, an impaired innate and adaptive immune response to influenza H1N1 combined with disrupted epithelial barrier function could lead to viral persistence in the lung. The observed para-cellular gaps and a decrease in TEER after cigarette smoke presumably facilitate the viral spread in the patient and lead to pro-longed infection.

In conclusion, our *in vitro* studies showed that cigarette smoke *per se* leads to suppression of pro-inflammatory cytokine and chemokine response of epithelial cells and—most importantly—also impairs antiviral and T cell cytokines after H1N1 infection. The observation that inflammation with subsets of cDCs and inflammatory DCs are increased *in vivo* goes along with the manifestation of respiratory inflammation, a process that is at the beginning not driven by an increased inflammatory response of epithelial cells. Thus, further studies are required to delineate the mechanistic link between smoke induced immunosuppression of epithelial cells and an increased number in DC populations after viral infection in CS-exposed mice. Overall, it can be speculated that inflammation after smoking is a secondary process as initially CS exposure disrupts the epithelial barrier and impairs antiviral immune response.

## Data Availability Statement

All datasets generated for study are included in the article/Supplementary Material.

## Ethics Statement

The animal study was reviewed and approved by Ministry of Energy, Agriculture, the Environment, Nature and Digitalization, Schleswig-Holstein, Germany; V 242 – 41093/2016 (82-7/16). Experiments with human lung tissue were approved by the Ethics Committee of the Hannover Medical School (MHH, Hannover, Germany) and are in compliance with The Code of Ethics of the World Medical Association (renewed on 2015/04/22, number 2701-2015). All patients or their next of kin gave written informed consent for the use of lung tissue for research.

## Author Contributions

OD, MW, SK-E, and KS wrote the original draft. OD, SBö, and HO performed the experiments with Calu-3 cells and PCLS. OD, KS, and SW conceived and designed the *in vitro* and *ex vivo* studies. DJ prepared lung tissue for PCLS generation. MW, BH, DK, and SR performed the experiments with mice. SK-E and SR conceived and designed the *in vivo* studies. MW, BH, SR, OD, and SBö performed data analysis and statistics. All authors reviewed and contributed to the final version of the manuscript.

## Conflict of Interest

The authors declare that the research was conducted in the absence of any commercial or financial relationships that could be construed as a potential conflict of interest.

## Abbreviations

ALI: Air-liquid interface
BALF: Bronchoalveolar lavage fluid
cDC: Conventional dendritic cell
COPD: Chronic obstructive pulmonary disease
CS: Cigarette smoke
DC: Dendritic cell
DMEM: Dulbecco's minimal essential medium
FBS: Fetal bovine serum
ffu: Focus forming units
Influenza H1N1: Influenza A/California/04/2009/H1N1/pandemic
IFN: Interferon
IL: Interleukin
IP-10: Interferon-γ-induced protein 10
I-TAC: Interferon-inducible T cell alpha chemoattractant
LDH: Lactatdehydrogenase
MCP: Monocyte chemoattractant protein
MHCII: Major histocompatibility complex class II
MSD: Meso Scale Discovery
NK: Natural killer
PBS: Phosphate buffered saline
PCLS: Precision-cut lung slice
pDC: Plasmacytoid dendritic cell
Poly (I:C): Polyinosinic–polycytidylic acid
TEER: Trans-epithelial electrical resistance
TLR: Toll-like receptor
TNF: Tumor necrosis factor
TPCK: N-tosyl-L-phenylalanine chloromethyl ketone.
