# Supplementary material for: Cigarette Smoke Affects Dendritic Cell Populations, Epithelial Barrier Function, and the Immune Response to Viral Infection With H1N1
Source: Front Med (Lausanne). 2020 Nov 6;7:571003. doi: 10.3389/fmed.2020.571003 (PMC7678748; doi:10.3389/fmed.2020.571003)
Supplement: Supplementary file 1 [file Data_Sheet_1.docx]

Supplementary Material

**Lung Function Assessment**

After intraperitoneal (i.p.) anesthesia (Narcoren, Merial, Germany), mice were tracheostomized and connected to the FlexiVent ventilator. (FlexiVent, SCIREQ, Canada). All perturbations (Single frequency forced oscillation (SnapShot-150), broadband frequency forced oscillation (Prime-8) and recruitment maneuver (Deep Inflation)) were performed one-by-one until three acceptable measurements (coefficient of determination >0.95) were recorded in each individual subject. To assess airway hyperreactivity, methacholine (MCh) (Sigma-Aldrich, Germany) was nebulized at increasing concentrations (6.25, 12.5, 25, 50 and 100 mg/ml) (Aeroneb Pro nebulizer, 3.1 µm MMAD; Aerogen Ltd, Ireland) and measurements were performed until a maximum was reached. After the last measurement final euthanasia was performed by cutting the abdominal aorta and blood was collected.

| 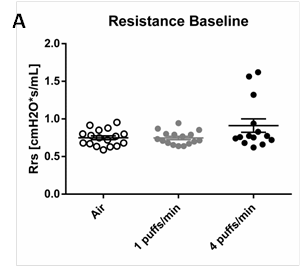 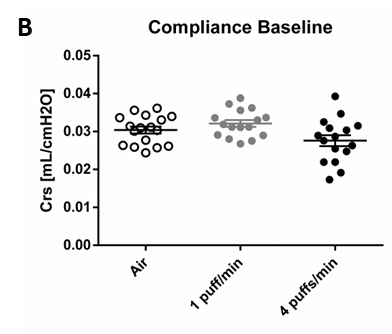 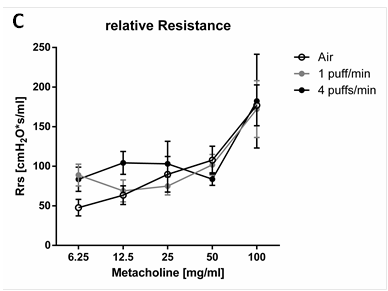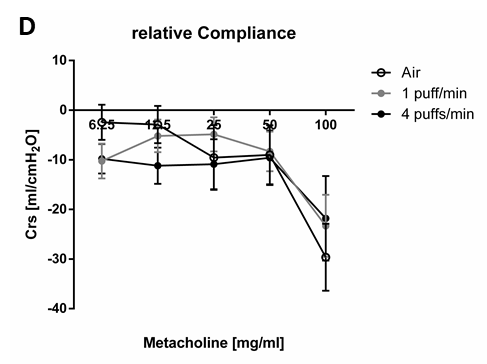 |
| --- |
| Supplementary Figure 1. Dose finding for smoking in mice. Lung function measurements of (A) baseline resistance and (B) baseline compliance among groups. (C) Relative resistance and (D) relative compliance at different methacholine concentrations (6.25, 12.5, 25, 50 and 100 mg/ml). (A-B) Data are expressed as mean ± SD, each data point represents an individual animal. (C-D) Data are expressed as mean ± SEM. (A-B) one way ANOVA. (C-D) 2way ANOVA. (A) Dunn’s multiple comparison test. (B-D) Tukey’s multiple comparison test.; room air (RA), bronchoalveolar lavage fluid (BALF). * p<0.05, ** p<0.01, *** p<0.001, **** p<0.0001. n = 10-16 animals per group in three independent experiments |

**Lung preparation for histology**

After rinsing the blood system, the left lung was fixed with 20% formaldehyde at a constant hydrostatic pressure of 20 cm H2O for 20 min. The sample was then dehydrated and embedded in paraffin, followed by staining of the tissue sections with PAS to assess histopathology.

| 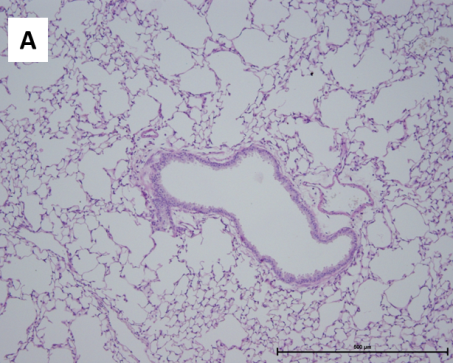 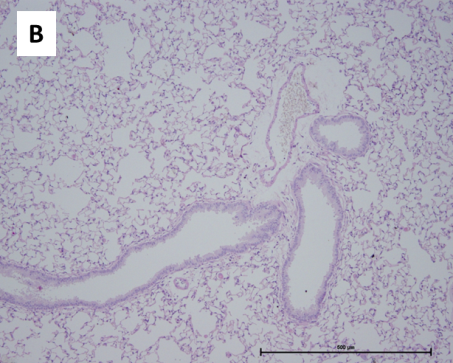 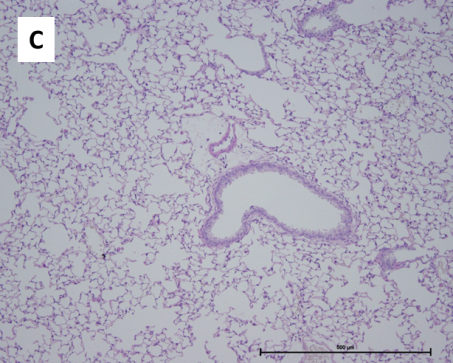 |
| --- |
| **Supplementary Figure 2. Cigarette smoke had no effect on histology of lungs** (A) lung of air control, (B) 1 puff/min and (C) 4 puffs/min exposed female C57Bl/6J. 10x magnification. |

## Flow Cytometry

Single cell suspensions were treated with Fc Block (CD16/CD32) against non-specific binding of antibodies on the cell surface. Subsequently, surface markers were labelled with fluorochrome-conjugated antibodies (supplementary Table 1).

**Supplementary Table 1.** Fluorochrome-conjugated antibodies for flow cytometry analysis

| Epitope | Fluorocrome | Clone | Manufacture | Country |
| --- | --- | --- | --- | --- |
| anti-MHCII | FITC | M5/114.15.2 | eBioscience | San Diego (USA) |
| anti-CD103 | PE | M290 | BD Bioscience | San Jose (USA) |
| anti-CD11b | PercP-Cy5.5 | M1/70 | BD Bioscience | San Jose (USA) |
| anti-CD11c | PE-Cy7 | HL3 | BD Bioscience | San Jose (USA) |
| anti-B220 | APC-Cy7 | PA3-6B2 | BioLegend | San Diego (USA) |
| anti-Ly6C | V450 | AL-21 | BD Bioscience | San Jose (USA) |

Fluorescence intensity was measured on a BD LSRII flow cytometer (BD Bioscience, USA) using the BD FACSDiva software. The excitation was performed with a 405 nm, 488 nm and 633 nm laser. The analyses were performed with FCS express Version 10 (FlowJo, LCC Software, USA). Percentage of cell counts was adjusted to previous counts of total single cell suspension. For gating strategy, see Supplementary Figure 2.

| 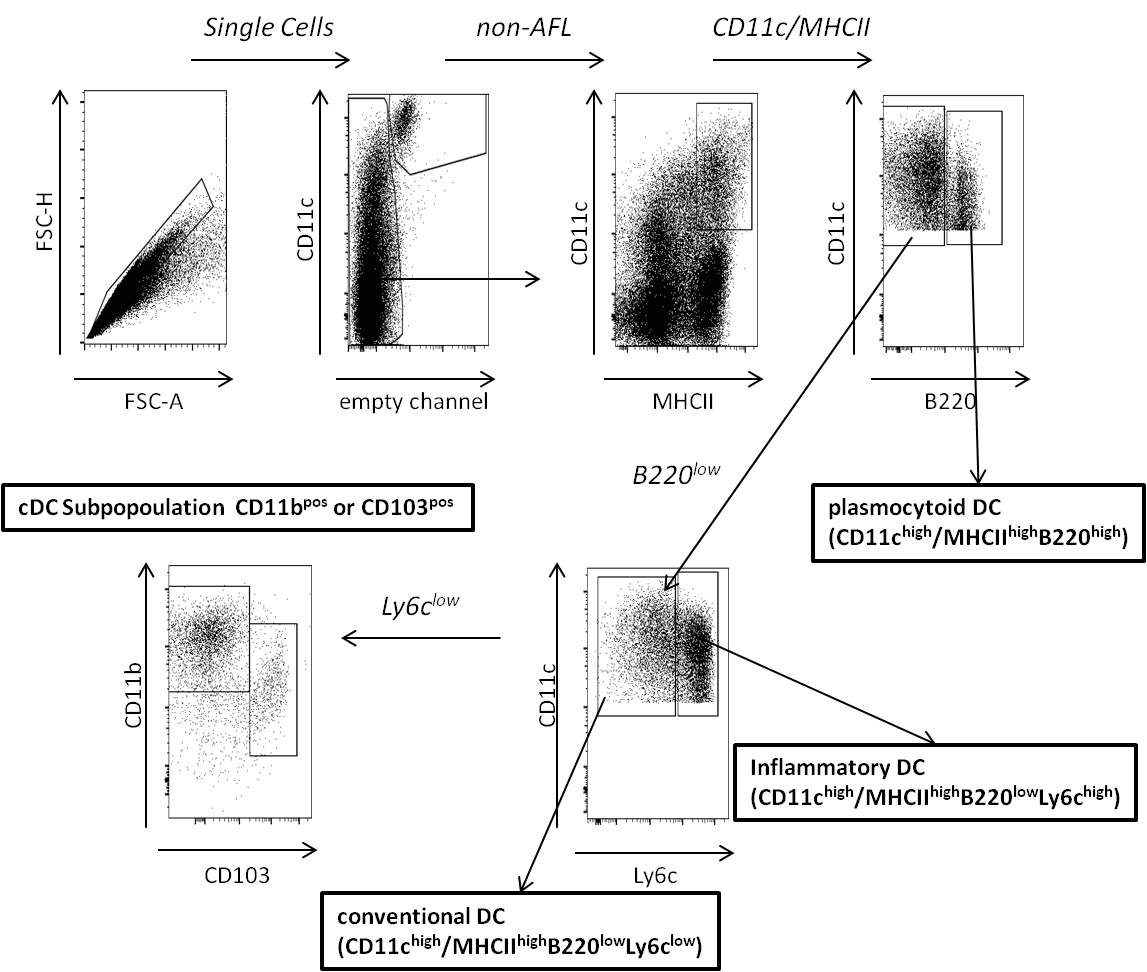 |
| --- |
| **Supplementary Figure 3.** Gating strategy for the differentiation of cells types by their labeled surface marker. |

**RNA Isolation**

Lung tissue was processed with a bead-based tissue homogenization method for 2 min at 30 Hz (TissueLyser II, Qiagen, The Netherlands). For lungs, total RNA including small RNAs was isolated using the miRNeasy Mini Kit (Qiagen, The Netherlands) according to the manufacturer’s protocol. RNA quality and quantity were controlled via spectrometry (DeNovix DS-11, DeNovix Inc., USA), standard gel electrophoresis. Only high-quality RNA samples (260/280 ratio> 1.66, no degradation as detected by capillary electrophoresis) were used for further analyses.

**qRT-PCR**

cDNA was transcribed using QuantiTect Reverse Transcription Kit, (Qiagen, the Netherlands) according to manufacturer’s protocol. Quantitative real-time PCR (qRT-PCR) was performed on a LightCycler 480 system (Roche Diagnostics, Germany) following denaturation for 10 min at 95 °C and 45 cycles with 10 s at 95 °C, 15 s at 60 °C, 10 s 72 °C and 1 s 78 °C. Dose finding data were normalized with the housekeeping genes HPRT and Tbp. The 2-ΔΔCt value was used to indicate the fold change in mRNA expression relative to air controls. Primers were generated by metabiom, (Germany):

*HPRT* sense (CAGGCCAGACTTTGTTGGAT), anti-sense (ACGTGATTCAAATCCCTGAAGT); *Tbp* sense (AATTGTACCGCAGCTTCAAAAT), anti-sense (ATGATGACTGCAGCAAATCG); *Cyp1a1* sense (CGTTACCTGCCTAACTCTTC), anti-sense (ATGCTCAATGAGGCTGTCTG).

**Treatment scheme**

| 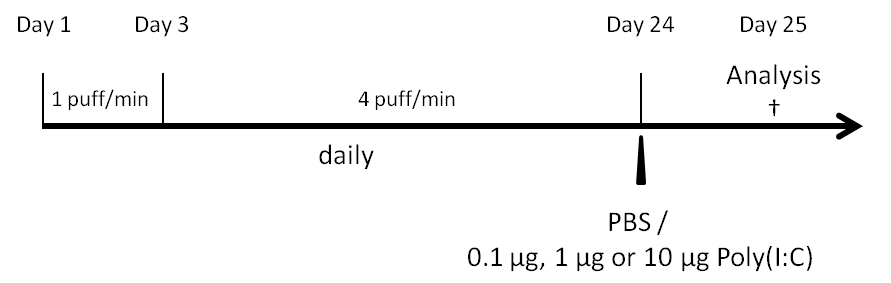 |
| --- |
| **Supplementary Figure 4.** Treatment scheme for exposure and intranasal application of mice. |

**UV-inactivated H1N1 did not induce immune response**

To analyze whether H1N1 induced immune response was due to active infection and replication, human PCLS were inoculated with UV-inactivated and thus replication-deficient H1N1 under submerse conditions. No induction of UV-H1N1-mediated immune response could be observed in lung tissue and confirmed specific immune response due to active virus infection and replication.


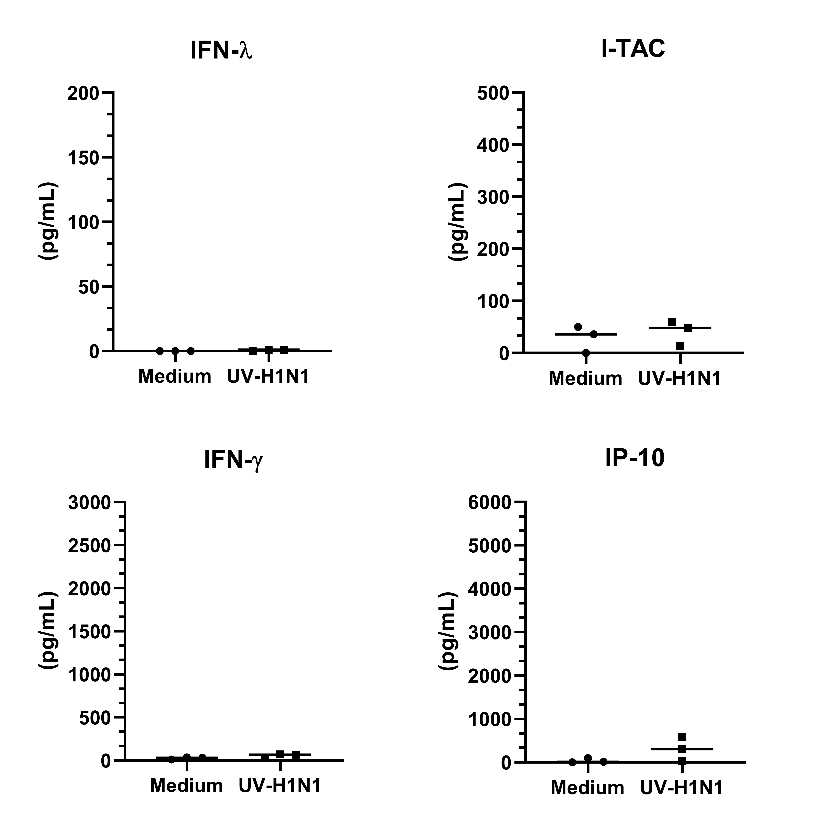


**Supplementary Figure 5.** UV-inactivated H1N1 did not induce immune response in human PCLS. Human PCLS were inoculated with influenza H1N1 (25,000 ffu/well) for 1 h and after inoculum has been removed post-incubated for 48 h. Cytokines were analyzed by ELISA or MSD. Every symbol represents an independent donor, with two technical replicates each, for n= 3 donors.

**Influenza H1N1 induced TNF-α release**

| 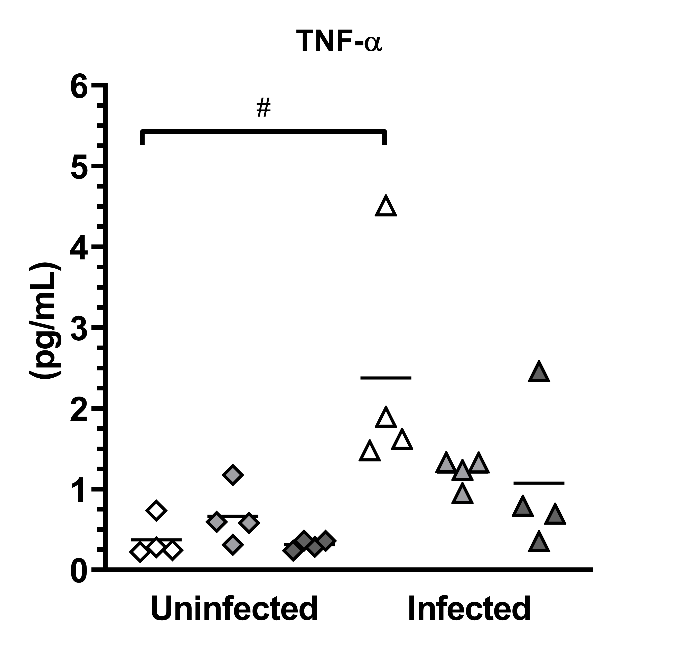 |  |
| --- | --- |


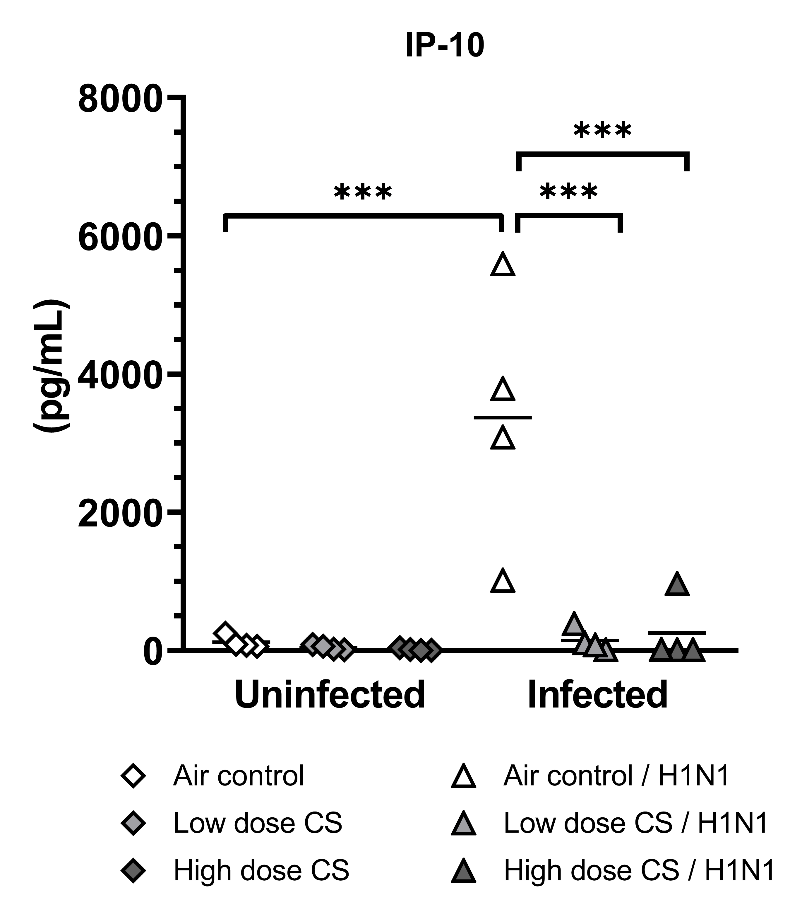


**Supplementary Figure 6.** CS impaired TNF-α release in H1N1-infected PCLS. Human PCLS were exposed to either air or two doses of CS at the ALI. Following exposure, the PCLS were post-incubated for 24 h and then infected with influenza H1N1 (25,000 ffu/well) at the apical surface. TNF-α release was induced in air-exposed and H1N1 infected PCLS but was inhibited in cigarette smoke exposed PCLS 48 h post infection. Every symbol represents an independent donor. n = 4 with four technical replicates each, # p < 0.05 air control vs. air infected control, analyzed by Tukey’s multiple comparisons test.
